# Potential bias and misclassification of using continuous cardiac output to identify fluid responsiveness compared to calibrated measurements

**DOI:** 10.1186/s13054-024-04993-1

**Published:** 2024-06-20

**Authors:** Laurent Bitker, Guillaume Deniel, Jean-Christophe Richard

**Affiliations:** 1 Service de Médecine Intensive – Réanimation, Hôpital de La Croix Rousse, Hospices Civils de Lyon, 103, Grande Rue de La Croix Rousse, 69004 Lyon, France; 2 Univ Lyon, Université Claude Bernard Lyon 1, INSA-Lyon, CNRS, INSERM, CREATIS UMR, 5220, U1294 Villeurbanne, France; 3 Université de Lyon, Université Claude Bernard Lyon 1, Villeurbanne, France

## Introduction

Fluid responsiveness was cornered as being of preeminent importance to optimize hemodynamics during circulatory shock [1]. This is facilitated in clinical routine by continuous cardiac output (CCO) monitoring. Yet, a theoretical risk exists of misclassification of fluid responsiveness if one uses the CCO value measured at the end of a fluid challenge (FC) without recalibrating the device. We hence evaluated the bias existing between calibrated cardiac output (CO) measured at FC’s end and the value of CCO measured immediately before that same re-calibration.

## Materials and methods

We report an ancillary of an observational single-center study performed in a tertiary ICU in Lyon, France. The study was approved by an ethics comity (Comité Scientifique et Ethique des Hospices Civils de Lyon, reference 23–5040). We enrolled consecutive patients with circulatory shock receiving norepinephrine and calibrated CCO monitoring (PiCCO®, Pulsion Medical, Germany), and who received a 500-ml FC of crystalloids in less than 15 min.

The primary outcome was the bias between the recalibrated CO (method 1) and the CCO value measured immediately at FC’s end and prior to the device recalibration (method 2). Secondary outcomes evaluated the trending and diagnostic performance of method 2 to identify fluid responsiveness.

The calibration was performed by mean of transpulmonary thermodilution (TPTD, 3 × injections of 15-ml cold saline). The device was calibrated twice, immediately before (T1) and immediately at FC’s end (T2, recalibration) to obtain calibrated CO (CO_TPTD_). CCO by pulse contour analysis was collected twice (continuous recording of the 12-s moving average of beat-to-beat CCO refreshed and sampled at 1 Hz): immediately after T1 (mean value over a 60-s stable hemodynamic period) and immediately before T2 (mean value over the last 60 s). We computed the respective relative changes in CCO (∆%CCO) and CO_TPTD_ (∆%CO_TPTD_) between T1 and T2. Fluid responsiveness was adjudicated if ∆%CO_TPTD_ increased > 15%.

Data were reported by their median [interquartile range], mean ± standard deviation, or count (percentage). Bias was evaluated using a Bland–Altman representation. Ability of ∆%CCO to track changes in ∆%CO_TPTD_ was assessed using 4-quadrant and radial plots. The diagnostic performance of ∆%CCO to identify fluid responsiveness was assessed using the area under the receiver operating curve (AUROC). 95% confidence intervals (_95%_c.i.) were computed using bootstrapping (n = 1000).

## Results

Between April 4th, 2023 and June 16th, 2023, we enrolled 15 patients within a delay of 1 [0.5–1] day after ICU admission (Supplemental Table 1 for the population characteristics). The elapsed time between T1 and T2 was 18 [14–20] min, and FCs were administered over 7 [7–8] min.

**Fig. 1:**
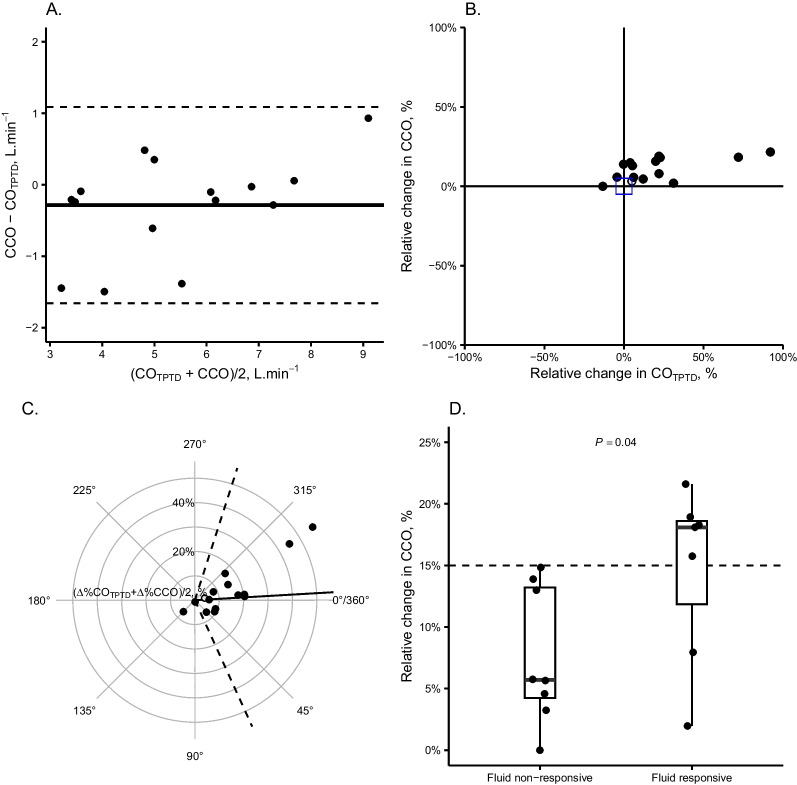
The figure shows the bias between CCO and CO_TPTD_ measured at the end of a fluid challenge (panel **A**), the concordance between the relative change in CCO (∆%CCO) and CO_TPTD_ (∆%CO_TPTD_) during the fluid challenge (panel **B**), the radial plot quantifying the bias in relative change (panel **C**) and the difference in relative change in CCO during the fluid challenge in patients identified as being fluid responders or non-responders (classified using a ∆%CO_TPTD_ threshold > 15%, panel **D**). Panel **A** is a Bland and Altman plot showing a constant bias (mean bias − 0.29 ± 0.70 L min^−1^, limits of agreements ± 1.4 L min^−1^). Panel **B** is a concordance plot, with concordant measurements situated in the north-eastern and south-western quadrants. Panel **C** is a radial plot showing the angular bias between ∆%CCO and ∆%CO_TPTD_, identified by the broad line, and the radial limits of agreement (dashed lines). The angular bias (− 3° ± 35°) was statistically different from 0° (*P* = 0.39), with radial limits of agreements of ± 71°. CCO: continuous cardiac output; CO_TPTD_: calibrated cardiac output by transpulmonary thermodilution; ∆%CCO: relative change in CCO between T1 and T2 (before the second calibration); ∆%CO_TPTD_: relative change in CO_TPTD_ between T1 et T2

The bias between methods at T2 was − 0.29 ± 0.70 L.min^−1^ (constant bias, limits of agreements ± 1.4 L.min^−1^). The CCO method had a percentage error of 25% (_95%_c.i.: 14%–36%) against CO_TPTD_ (Fig. 1A). ∆%CCO demonstrated intermediate trending ability to detect changes in ∆%CO_TPTD_ (Fig. 1B), with a concordance rate of 79% (_95%_c.i.: 49%–94%). The radial plot showed an angular bias between methods of − 3° ± 35° (Fig. 1C).

Fluid responsiveness was identified in 7/15 of FCs using CO_TPTD_. ∆%CCO was significantly higher in fluid responders compared to non-responders (Fig. 1D), although ∆%CCO was < 15% in 2 fluid-responsive cases. ∆%CCO had an AUROC of 0.83 (_95%_c.i.: 0.60–1.00, *P* = 0.04) to identify fluid responsiveness. At the threshold of 15%, ∆%CCO had a sensitivity of 0.70 (_95%_c.i.: 0.38–1.00) and a specificity > 0.99 (_95%_c.i.: 0.99–1.00) to identify fluid responsiveness.

## Discussion and conclusion

In this single-center observational study, we identified that 1/ CCO measured immediately before CO recalibration after a FC demonstrated a small negative bias; 2/ ∆%CCO demonstrated intermediate trending capacity with potentially large bias between methods; and 3/ ∆%CCO had acceptable classifying performance to identify fluid responsiveness, with a risk of false negative results.

Our findings suggest that, while performing a FC monitored by calibrated CCO, cautious interpretation of the FC’s results should be made, due to potential bias impacting its relative change from baseline. The pharmacokinetics of a FC show that the infusion of 500 ml of crystalloid at 20 °C may not only improve venous return and potentially CO, but could also alter arterial or venous compliance and resistance [2]. These modifications will eventually modify the arterial root signal of CCO, and lead to misclassification [3].

FC’s hemodynamic effect dissipation occurring between the end of the FC and the end of recalibration (∽5 min) may not be retained, as the bias between method was negative (i.e. CCO was lower than CO_TPTD_), and no cases showed a ∆%CCO > 15% in non-responders [4]. Finally, CO_TPTD_ measured by triplicate injection demonstrates a precision of ∽7% and least significant change (LSC) of ∽10%, which implies potentially inaccurate adjudication of fluid responsiveness using this technique [5].

To conclude, using CCO to evaluate fluid responsiveness in patients receiving a FC has the advantage of being efficient, but goes with the risk of misclassification and misleading clinical conclusions.

### Supplementary Information

Supplementary file 1.

## Data Availability

Source datasets are not publicly available due to ethical reasons. Further enquiries can be directed to the corresponding author.

## Abbreviations

AUROC: Area under the receiver-operating curve
CCO: Continuous cardiac output
CO_TPTD_: Calibrated cardiac output by transpulmonary thermodilution
∆%CCO: Relative change in CCO between T1 and T2 (before the second calibration)
∆%CO_TPTD_: Relative change in CO_TPTD_ between T1 et T2
FC: Fluid challenge
TPTD: Transpulmonary thermodilution

## References

[1] Cecconi M, De Backer D, Antonelli M, Beale R, Bakker J, Hofer C, Jaeschke R, Mebazaa A, Pinsky MR, Teboul JL (2014). Consensus on circulatory shock and hemodynamic monitoring task force of the european society of intensive care medicine. Intensive Care Med.

[2] Bitker L, Cutuli SL, Yanase F, Wilson A, Osawa EA, Lucchetta L, Cioccari L, Canet E, Glassford N, Eastwood GM (2022). The hemodynamic effects of warm versus room-temperature crystalloid fluid bolus therapy in post-cardiac surgery patients. Perfusion.

[3] Hamzaoui O, Monnet X, Richard C, Osman D, Chemla D, Teboul JL (2008). Effects of changes in vascular tone on the agreement between pulse contour and transpulmonary thermodilution cardiac output measurements within an up to 6-hour calibration-free period. Crit Care Med.

[4] Aya HD, Ster IC, Fletcher N, Grounds RM, Rhodes A, Cecconi M (2016). Pharmacodynamic analysis of a fluid challenge. Crit Care Med.

[5] Giraud R, Siegenthaler N, Merlani P, Bendjelid K (2017). Reproducibility of transpulmonary thermodilution cardiac output measurements in clinical practice: a systematic review. J Clin Monit Comput.

