# Supplementary material for: Potential bias and misclassification of using continuous cardiac output to identify fluid responsiveness compared to calibrated measurements
Source: Crit Care. 2024 Jun 20;28:202. doi: 10.1186/s13054-024-04993-1 (PMC11188275; doi:10.1186/s13054-024-04993-1)
Supplement: Supplementary file 1 [file 13054_2024_4993_MOESM1_ESM.docx]

| **Table 1.** Baseline characteristics of the population at inclusion | |
| --- | --- |
|  | N=15 |
| Age, years | 72 [58–76] |
| Gender, male, N (%) | 12 (80%) |
| BMI, kg.m^-2^ | 25 [19–30] |
| Admission category, medical, N (%) | 14 (93%) |
| *Severity of disease* |  |
| SAPS-2 score at ICU admission | 60 [50–72] |
| SOFA score on inclusion day | 9 [8–12] |
| Sepsis on inclusion day, N (%) | 11 (73%) |
| Septic shock on inclusion day, N (%) | 10 (67%) |
| Invasive mechanical ventilation, N (%) |  |
| Lowest PaO_2_/FiO_2_ ratio on day of inclusion, mmHg | 235 [185–304] |
| ARDS, N (%) | 4 (27%) |
| Richmond Analgesia and Sedation Scale (RASS) | -4 [-5–0] |
| *Cardiac rythm* |  |
| Sinus, N (%) | 12 (80%) |
| *Hemodynamics* |  |
| Heart rate, min^-1^ | 83 [72–110] |
| Mean arterial pressure, mm Hg | 79 [72–84] |
| Central venous pressure, mm Hg | 6 [4–8] |
| CCO, L.min^-1^ | 4.7 [3.7–5.7] |
| CO_TPTD_, L.min^-1^ | 4.7 [3.6–5.9] |
| SVI, mL.m^-2^ | 29 [24–36] |
| GEDVI, mL.m^-2^ | 680 [545–866] |
| EVLWI, mL.kg^-1^ PBW | 8.5 [6.9–11.2] |
| Arterial lactate, mmol.L^-1^ | 2.1 [1.4–2.7] |
| Norepinephrine dose (tartrate base), µg.kg^-1^.min^-1^ | 0.20 [0.06–0.45] |
| Data are presented as count (%) or median [interquartile range].  ARDS: acute respiratory distress syndrome; BMI: body mass index; CCI_PCA_: continuous cardiac index measured by pulse contour analysis; CI_TPTD_: cardiac index measured by transpulmonary thermodilution; EVLWI: extravascular lung water index; FiO_2_: inspired fraction in O_2_; GEDVI: global end-diastolic volume index; ICU: intensive care unit; PaO_2_: arterial partial pressure in O_2_; PBW: predicted body weight; PEEP: positive end-expiratory pressure; SAPS-2: simplified acute physiology score 2; SOFA: sepsis-related organ failure assessment; SVI: stroke volume index | |
